# Editorial: Developing novel materials and new techniques of biological and physical retrospective dosimetry for affected individuals in radiological and nuclear emergencies

**DOI:** 10.3389/fpubh.2022.1117269

**Published:** 2023-01-05

**Authors:** Nadica Maltar-Strmečki, Emanuela Bortolin, Laura Kenzhina, Clarice Patrono, Antonella Testa

**Affiliations:** ^1^Laboratory for Electron Spin Resonance, Division of Physical Chemistry, Ruđer Bošković Institute (RBI), Zagreb, Croatia; ^2^Core Facilities, Istituto Superiore di Sanità (ISS), Rome, Italy; ^3^Institute of Radiation Safety and Ecology (IRSE), National Nuclear Center of Kazakhstan, Kurchatov, Kazakhstan; ^4^Italian National Agency for New Technologies, Energy and Sustainable Economic Development (ENEA), Rome, Italy

**Keywords:** radiological and nuclear emergencies, stimulated luminescence, electron spin resonance, biological dosimetry, emergency preparedness and response

Retrospective dosimetry estimates radiation doses received by an individual in the past using physical and biological methods. Because of the increasing threat of radiological accidents or terrorist attacks involving radioactive material, the development of this area of research has become extremely important (1, 2). This Research Topic, entitled “Developing Novel Materials and New Techniques of Biological and Physical Retrospective Dosimetry for Affected Individuals in Radiological and Nuclear Emergencies,” provided an opportunity to receive new relevant contributions from various experts who provided new insights and perspectives in the field of retrospective dosimetry. The aim was to connect more scientific fields, researchers and infrastructures from this interdisciplinary research area and to collect studies relevant to human health. The main objective is to improve complementary physical and biological measurement techniques with lower detection limits of received radiation doses, to characterize different appropriate dosimeters, to develop and validate individual dose estimation models, and to propose standardized dosimetry protocols required in the most likely scenarios. It also aims to estimate more accurately radiation dose received by individuals involved in a nuclear accident or radiological emergency using materials on or near the victim and the victim's blood in a relatively short period of time to allow appropriate medical treatment and therapy to increase survival rates.

This Research Topic consists of 7 articles (six original research articles and one brief research report) published in the Radiation and Health section of Frontiers in Public Health.

To date, physical methods of retrospective dosimetry have shown promising dosimetric properties for certain materials (glass, alanine, sugar, plastic, silicates, tobacco, etc.). However, for practical use, it is essential to characterize these and new materials and to lower the detection limits of the physical methods. In the event of a mass casualty incident, it is critical to have dosimeters capable of detecting and evaluating very low radiation doses, well below those used for clinical treatments, in order to quickly distinguish between those seriously exposed to radiation and the so-called “worried well” who are not exposed to significant radiation doses. Detection limits should be at levels at which health effects are possible but lower than the dose (8 Gy) at which survival is not expected. Particular attention has been given to the 1 Gy limit, which separates individuals requiring medical attention from those who are largely unaffected and do not require immediate treatment (1, 2).

Cell phone and watch glasses are attractive as incidental dosimeters because they are in close contact with humans. Therefore, they were studied using electron paramagnetic resonance (EPR) spectroscopy and stimulated luminescence methods of physical retrospective dosimetry. New insights into the method of dose reconstruction were provided in this Research Topic by Marciniak et al. Their study compared three methods of dose reconstruction based on EPR measurements of watch glass samples: the calibration method (CM), the added dose method (ADM), and the added dose and heating method (ADHM). The results showed that the three methods of dose reconstruction provide reliable and similar results in the dose range of 0.5–6.0 Gy, with an accuracy better than 10%. However, the ADHM method is the only one applicable in a real scenario when no sample-specific background spectrum is available. Because the background signal from the glass is a limitation in accurate dose reconstruction regardless of the method used - EPR or stimulated luminescence - Bassinet et al. proposed in their study the use of a chemical treatment with hydrofluoric acid (HF 40%) to minimize the contribution of the intrinsic thermoluminescence (TL) signal from the surface of the cell phone protector glass. A decrease was observed after an etching time of 1 min, but a detailed study of the glass composition is required to confirm these results. To reduce the contribution of the background, further tests were performed in the same work by enhancing the red component of the luminescence with special photomultipliers and filters. The preliminary results obtained are encouraging, but need to be verified by further investigations.

Complementary to the above research, interest in optically stimulated luminescence (OSL) has been rekindled due to the possibility of the fast readout without special sample preparation. As a result, various materials that can be found near or at the victim without much cost have been the focus of many recent studies. Alghamdi et al. compared the low detection limits achieved using household salt with portable OSL readers in terms of different stimulation wavelengths and different types of OSL devices. Their results confirmed the method as a rapid and effective for determining doses below 0.1 mGy. In addition to household salt, Ekendahl et al. studied the OSL response to irradiated common pharmaceuticals such as ibuprofen, paracetamol, aspirin, oral contraceptives, motion sickness prevention drugs, and food supplements such as table vitamins and minerals. Results suggest a high fading rate and dependence on MgO content, but feasible dose reconstruction for doses in the range of a few Gy when performed no later than 24–72 h after irradiation.

Biological dosimetry using standardized techniques currently requires fresh blood from potential victims and the establishment of cell cultures, which is a significant obstacle to rapid and immediate dose reconstruction (1, 2). In addition, most established tests are characterized by long scoring times at the microscope.

Over the past decade, the search for faster methods to accelerate dose assessment has increased. During large-scale radiological or nuclear incidents, a significant number of people are potentially exposed to excessive radiation doses. In this case, there is a need for rapid and simple biological dosimetry methods for initial triage to determine the level of radiation exposure and whether medical treatment is required. Quantification of radiation-induced DNA double-strand breaks (DSBs) by the accumulation of DSB repair proteins that can be labeled and counted is a useful tool for dose estimation. Currently, counting of γH2AX foci is used as a reliable and sensitive biological dosimetry method to detect exposure to very low doses of ionizing radiation in individuals.

Other DSB-repair proteins constitute promising markers for radiation-induced DSBs quantification and can be used for biological dose estimation.

In the manuscript by Chaurasia et al., the authors propose the detection and quantification of phospho-53BP1 foci as a rapid and sensitive biological dosimetry method. For this purpose, γ-ray-induced dose-response curves were generated and validated at different time points. The study also includes comparative evaluations of the generated calibration curve with the γH2AX-foci assay and cytogenetic assays by estimating doses from dose-blinded samples. The authors suggest the use of the phospho-53BP1 foci calibration curve for rapid biological dosimetry in radiological emergencies and medical exposures.

Another system to reduce analysis time is the use of automated or semi-automated equipment. Automation of metaphase determination, image capturing, and aberration scoring has proven successful and is used in many biodosimetry laboratories. In this context, an interesting work by Lee et al. has been incorporated into this Research Topic. In their study, the performance of a semi-automatic scoring method for dicentrics using *ex vivo* and *in vivo* irradiated samples was compared to the standard manual scoring method. Although the semi-automated scoring method requires additional testing to correct for false positives, their results indicate that the semi-automated scoring method can rapidly provide an accurate dose estimate and could be useful as an alternative to the manual dicentric assay for bio-dosimetry in large-scale radiological accidents.

In addition, Buchner et al. indicated in their study that the orientation of the blood sample tubes (vertical vs. horizontal) had a significant effect on radiation dose with a variation from −41 to +49% and contributed to a dose gradient of up to 870 mGy within the vertical tubes due to the size of the sample tubes and the associated differences in the distance from the focal point of the tube. As a result, the number of dicentric chromosomes and micronuclei differed by ~30% between the two orientations. Therefore, careful examination of the experimental setup in collaboration with physicists was suggested to ensure traceability and reproducibility of the irradiation conditions, to correctly correlate the radiation dose and the number of aberrations, and to avoid systematic biases that affect dose estimation in the context of biological dosimetry.

According to these findings, it can be suggested as a conclusion that close cooperation between a wide range of researchers and infrastructures is needed to achieve the main goal of urgent, appropriate and adequate medical treatment of victims of radiological and nuclear accidents.

The Editors of this Research Topic thank the distinguished Authors for their invaluable contributions and are indebted to the expert Reviewers for their time, dedication, and constructive comments. We thank the Radiation and Health Section of Frontiers in Public Health for the opportunity to guest edit this Research Topic.

## Author contributions

All authors listed have made a substantial, direct, and intellectual contribution to the work and approved it for publication.
